# Benchmarking calf health: Assessment tools for dairy herd health consultancy based on reference values from 730 German dairies with respect to seasonal, farm type, and herd size effects

**DOI:** 10.3389/fvets.2022.990798

**Published:** 2022-09-23

**Authors:** Linda Dachrodt, Alexander Bartel, Heidi Arndt, Laura Maria Kellermann, Annegret Stock, Maria Volkmann, Andreas Robert Boeker, Katrin Birnstiel, Phuong Do Duc, Marcus Klawitter, Philip Paul, Alexander Stoll, Svenja Woudstra, Gabriela Knubben-Schweizer, Kerstin Elisabeth Müller, Martina Hoedemaker

**Affiliations:** ^1^Clinic for Cattle, University of Veterinary Medicine Hannover, Foundation, Hannover, Germany; ^2^Department of Veterinary Medicine, Institute for Veterinary Epidemiology and Biostatistics, Freie Universität Berlin, Berlin, Germany; ^3^Behavioral Physiology of Livestock, Institute of Animal Science, University of Hohenheim, Stuttgart, Germany; ^4^Clinic for Ruminants With Ambulatory and Herd Health Services, Centre for Clinical Veterinary Medicine, Oberschleissheim, Germany; ^5^Department of Veterinary Medicine, Clinic for Ruminants and Swine, Freie Universität Berlin, Berlin, Germany; ^6^VetZ GmbH, Isernhagen, Germany

**Keywords:** diarrhea, bovine respiratory disease, omphalitis, organic farming, benchmarking tool, animal wellbeing and welfare, calf disease

## Abstract

Good calf health is crucial for a successfully operating farm business and animal welfare on dairy farms. To evaluate calf health on farms and to identify potential problem areas, benchmarking tools can be used by farmers, herd managers, veterinarians, and other advisory persons in the field. However, for calves, benchmarking tools are not yet widely established in practice. This study provides hands-on application for on-farm benchmarking of calf health. Reference values were generated from a large dataset of the “PraeRi” study, including 730 dairy farms with a total of 13,658 examined preweaned dairy calves. At herd level, omphalitis (O, median 15.9%) was the most common disorder, followed by diarrhea (D, 15.4%) and respiratory disease (RD, 2.9%). Abnormal weight bearing (AWB) was rarely detected (median, 0.0%). Calves with symptoms of more than one disorder at the same time (multimorbidity, M) were observed with a prevalence of 2.3%. The enrolled farms varied in herd size, farm operating systems, and management practices and thus represented a wide diversity in dairy farming, enabling a comparison with similar managed farms in Germany and beyond. To ensure comparability of the data in practice, the reference values were calculated for the whole data set, clustered according to farm size (1–40 dairy cows (*n* = 130), 41–60 dairy cows (*n* = 99), 61–120 dairy cows (*n* = 180), 121–240 dairy cows (*n* = 119) and farms with more than 240 dairy cows (*n* = 138), farm operating systems (conventional (*n* = 666), organic (*n* = 64)) and month of the year of the farm visit. There was a slight tendency for smaller farms to have a lower prevalence of disorders. A statistically significant herd-size effect was detected for RD (*p* = 0.008) and D (*p* < 0.001). For practical application of these reference values, tables, diagrams, and an Excel^®^ (Microsoft^®^) based calf health calculator were developed as tools for on-farm benchmarking (https://doi.org/10.6084/m9.figshare.c.6172753). In addition, this study provides a detailed description of the colostrum, feeding and housing management of preweaned calves in German dairy farms of different herd sizes and farm type (e.g., conventional and organic).

## Introduction

The most common disorders in preweaned dairy calves are diarrhea, respiratory disease, and omphalitis (1). Diseases in calves have a variety of negative effects such as growth retardation, a higher susceptibility to develop further diseases and an increased risk of mortality (2–5). A wide spectrum of risk factors affecting calf health have been reported, including energy supply of the dam (6), colostrum supply of the neonate (7), housing conditions (8), and plane of nutrition (9). Previous studies have shown that farm-specific characteristics, e.g., colostrum management (10) and housing conditions (11), are closely related to region and herd size. Season (12), climate (13, 14), number of dairy cows (15), farm type [organic, conventional, (16)], and region also have a great impact.

The health status of its youngstock substantially contributes to the profitability of a dairy farm. Therefore, on-farm monitoring of health indicators should form an integral part of the routine work on dairy farms. Currently, the choice of appropriate health indicators, and the classification of the results obtained with respect to the quality indicate no uniformity among the persons involved in the calf rearing process. Likewise, there is a degree of farm blindness regarding poor conditions (17). For this reason, objective assessment parameters are needed to evaluate the health status of preweaned dairy calves during the farm visit. Benchmarking is a simple established method initially used in industry for comparing the performance of producers with respect to product quality and has been introduced in dairy farming. Benchmarking enables a comparison of farms sharing similar characteristics and simultaneously helps to identify areas for potential improvement (18, 19).

In modern dairy farming, a wide range of sensor data from lactating dairy cows, e.g., milk yield and udder health indicators (20) or indicators of fertility (21), are already systematically collected and analyzed in the daily work routine. Previous studies have already shown that farmers who have access to data related to calf health from other farms are highly motivated to improve their own management practices, e.g., by aiming at increasing average daily weight gains of their preweaned dairy calves (22, 23). In addition, the use of benchmarks can help to reinforce the relationship between farmers and veterinarians (24). In this context, it was also found that farmers motivated by a trusted advisor were more likely to make changes in disease prevention management (25).

Despite the already observed positive effects of benchmarking, to date, there are no widely established hands-on applications available to assess the health status of preweaned calves on farms. There is also a lack of reference values on herd-level for prevalence data on diseases in preweaned dairy calves based on a large study population. The scarce available literature on calf health on organic farms does not yet provide representative data that can be made applicable to all organic farms (26). Furthermore, most studies focus on conventional farms. Therefore, the aim of the present study was to use a large and diverse data set, including 730 German dairy farms and 13,658 examined preweaned dairy calves to provide representative herd level reference values in tables and figures for use in on-farm consultancy. A further aim was to develop a digital calf health calculator which allows farmers, herd managers, veterinarians, and other advisory persons to benchmark farm data on the basis of these reference values.

## Materials and methods

### Data set

As part of the prevalence study “PraeRi” (25), 731 farms in three regions of Germany with intensive dairy farming were visited on a single occasion between December 2016 and July 2019. Farm visits included seven federal states: region north: Schleswig-Holstein (*n* = 64), Lower Saxony (*n* = 173); region east: Mecklenburg-Western Pomerania (*n* = 65), Brandenburg (*n* = 65), Thuringia (*n* = 46), Saxony-Anhalt (*n* = 71); region south: Bavaria (*n* = 247). The final data set included 730 farms with a total of 13,658 calves (one farm with one calf was excluded because information about fecal consistency was missing).

### Farm selection

In Bavaria, the farms were randomly selected by a neutral auditing organization for Bavarian dairy farms (Milchprüfring Bayern e.V.) and in the remaining federal states, the farms were randomly selected from the complete list of cattle owners in the Identification and Registration of Bovine Animals in accordance with Regulation (EC) No. 1760/2000 Germany (Herkunftssicherung- und Informationssystem für Tiere, HI-Tier). The selection was made using SAS software version 9.4 (SAS Institute Inc., Cary, NC, USA). The farms were officially invited by the local authorities or the Dairy Herd Improvement Association. The participation was voluntary. The participation rate varied among the three regions between 6 and 9%. In total, 8,944 farms were invited, and of these, 765 farms were visited. On the day of the farm visit, 731 farms kept preweaned dairy calves [details published elsewhere (1)].

### Training of veterinarians and interobserver comparison

The farm visits and clinical examination were performed by 21 veterinarians. The study veterinarians were employed exclusively for this study. A training session lasting several days was conducted before the study started and Standard Operation Procedures (SOP) were defined for the collection and analysis of the data. An interobserver comparison was performed once a year to ensure the quality of the collected data. Each observer received an individual evaluation of his/her achievements. When significant deviations were observed for individual observers, an individual problem analysis was performed [details concerning the Interobserver Reliability are published elsewhere (1)].

### Study population

The study population included calves, which received milk or milk replacer, aged 24 h to a maximum of 6 months. In total, a median of 14 (Min: 1; Max: 350) calves were present on the farms at the day of the visit (Supplementary Table 3). Depending on herd size, at least one to a maximum of 75 preweaned calves were clinically examined (Supplementary Table 3). In total, for almost all preweaned calves (96%, IQR: 83–100%) on farm an examination was performed (Table 1). Each calf was identified by the last five digits of its ear tag. Data on age, sex, and breed were collected from the online data bank HI-Tier (www.hi-tier.de). Overall, the median age of preweaned calves enrolled in the study was 37 (IQR: 16–62, Table 1) days. The most commonly reported breeds were Holstein Friesian (73.4%) and Simmental (12.7%). In total, one quarter of the examined calves were male [data published elsewhere (1)].

**Table 1:** Study population and farm data for 730 dairy farms in Germany stratified by herd size and farm type.

**No. farms (*n)***		**Median (IQR)** ^**#**^						
		**Conventional**						
	**Total (*n* = 730)**	**Overall (*n* = 666)**	1–40*(*n* = 130)	41–60*(*n* = 99)	61–120*(*n* = 180)	121–240*(*n* = 119)	>241*(*n* = 138)	**Organic (*n* = 64)**
**Study population**								
Dairy cows (*n*)	84 (44, 88)	90 (48, 206)	27 (22, 34)	51 (46, 57)	85 (69, 102)	162 (137, 204)	426 (317, 644)	42 (27, 80)
Preweaned calves (*n*)	13 (7, 27)	15 (8, 31)	5 (3, 8)	10 (7, 13)	13 (9, 18)	24 (17, 31)	66 (45, 90)	7 (3, 12)
Examined calves (*n*)	12 (6, 25)	13 (7, 28)	5 (3, 7)	9 (6, 12)	12 (8, 17)	21 (16, 29)	41 (37, 71)	6 (3, 11)
Examined calves (%)	96 (83, 100)	95 (82, 100)	100 (100, 100)	100 (86, 100)	100 (89, 100)	94 (87, 100)	79 (64, 89)	100 (89, 100)
Age at weaning (wk)	11 (10, 12)	11 (9, 12)	11 (9, 12)	12 (10, 12)	11 (9, 12)	11 (9, 12)	10 (10, 12)	12 (12, 14)
**Area under cultivation**								
Total area (ha)	100 (52, 328)	104 (55, 400)	32 (23, 50)	56 (43, 90)	92 (68, 120)	190 (130, 438)	1,300 (726, 2,000)	63 (36, 128)
Thereof grassland (ha)	44 (24, 100)	45 (24, 100)	18(12, 29)	25 (20, 35)	41(30, 64)	78 (50, 130)	280 (128, 455)	40 (26, 63)
Thereof arable (ha)	50 (19, 200)	55 (21, 260)	10 (3, 25)	30 (15, 50)	45 (66, 325)	140 (50, 130)	960 (487, 1,500)	24 (0, 78)

^#^Interquartile range.

*Number of dairy cows.

### Clustering by herd size and farm type

The 730 farms differed markedly in herd size, farm operating systems, and management. Depending on the number of dairy cows, the farms were assigned to one of five different herd size groups: 1–40 dairy cows (*n* = 130), 41–60 dairy cows (*n* = 99), 61–120 dairy cows (*n* = 180), 121–240 dairy cows (*n* = 119), and farms with more than 240 dairy cows (*n* = 138). Farms of different size varied regarding the area under cultivation, additional occupation of the farmers (full- or part-time business) and their use of veterinary herd health management (VHHM) advisory services (Tables 1, 2). Due to the structural differences between organic and conventional farms, a separate description of the organic farms was issued. The organic farms had a median of 41.5 (IQR: 27.0, 79.5) dairy cows, cultivated a median area of 63.0 hectares and were sometimes (18.8%) run as part-time businesses (Table 2).

**Table 2:** Farm organization and the use of veterinary herd health management (VHHM) advisory services on 730 dairy farms in Germany stratified by herd size and farm type.

		**N (%)**						
		**Conventional**						
	**Total (*n* = 730)**	**Overall (*n* = 666)**	1–40*(*n* = 130)	41–60*(*n* = 99)	61–120*(*n* = 180)	121–240*(*n* = 119)	>241*(*n* = 138)	Organic(*n* = 64*)
**Farm organization**								
Full-time business	676 (92.6)	624 (93.8)	90 (69.8)	98 (99.0)	179 (99.4)	119 (100.0)	138 (100.0)	52 (81.2)
Part-time business	54 (7.4)	41 (6.2)	40 (30.8)	1 (1.0)	1 (0.6)	0 (0.0)	0 (0.0)	12 (18.8)
Conventional	661 (90.4)	660 (99.1)	127 (97.7)	97 (98.0)	179 (99.4)	119 (100.0)	138 (100.0)	0 (0.0)
Organic	64 (8.8)	0 (0.0)	0 (0.0)	0 (0.0)	0 (0.0)	0 (0.0)	0 (0.0)	64 (100.0)
Transition^#^	6 (0.8)	6 (0.9)	3 (2.3)	2 (2.0)	1 (0.6)	0 (0.0)	0 (0.0)	0 (0.0)
**VHHM**								
For dairy cows	399 (54.7)	346 (52.0)	107 (82.3)	66 (66.7)	95 (53.1)	45 (37.8)	33 (23.9)	52 (82.5)
For youngstock	159 (21.8)	153 (23.0)	7 (5.3)	12 (12.2)	24 (13.4)	34 (28.6)	76 (55.1)	6 (9.4)

*Number of dairy cows.

^**#**^Farms in process of transition from conventional to organic farming (in the analysis they were evaluated as conventional farms).

The description of the farms (herd size, area under cultivation, farm type) in dependence on three regions (north, east, and south) is given in the Supplementary Tables 1, 2.

### Calf rearing strategies of farms enrolled in the “PraeRi” study

#### Colostrum management

On the day of the farm visit, an interview with the farmer (or herd manager) was conducted. Questionnaires were used to collect information on calving area (e.g., maternity pen, pen of lactating cows, pasture), colostrum (quantity, feeding strategy), and feeding management (milk or milk replacer, solid feed, water) of preweaned calves up to weaning. The majority of farms (74.2%) fed colostrum with a teat bucket (Table 3). In just over a fifth of the farms (21.4%) calves sucked the dam as colostrum feeding strategy. This mainly concerned farms with 61–120 dairy cows (27.8%) and farms with 121–240 dairy cows (26.9%, Table 3). In more than two-thirds of the farms (68.6%), up to 3 l of colostrum were offered (Table 3). Overall, 26.6% of the farms offered 3–4 l of colostrum (Table 3). More than 4 l were rarely fed (4.8%); the highest proportion of farms offering more than 4 l of colostrum were found in the herd size group with more than 240 dairy cows (8.2%, Table 3).

**Table 3:** Colostrum management in 730 dairy farms in Germany stratified by herd size and farm type.

		***N*** **(%)**						
		**Conventional**						
	**Total (*n* = 730)**	**Overall (*n* = 666)**	1–40*(*n* = 130)	41–60*(*n* = 99)	61–120*(*n* = 180)	121–240*(*n* = 119)	>241*(*n* = 138)	Organic (*n* = 64*)
**Supply**								
Sucking the dam	156 (21.4)	129 (19.4)	12 (9.2)	21 (21.2)	50 (27.8)	32 (26.9)	14 (10.3)	27 (42.2)
Bucket feeding	541 (74.2)	504 (75.9)	118 (90.8)	78 (78.8)	123 (68.3)	81 (68.1)	104 (76.5)	36 (56.2)
Esophageal tube	31 (4.3)	30 (4.5)	0 (0.0)	0 (0.0)	6 (3.3)	6 (5.0)	18 (13.2)	1 (1.6)
**Quantity**								
Up to 3 liters	397 (68.6)	374 (69.3)	93 (78.2)	59 (75.6)	87 (65.9)	61 (68.5)	74 (60.7)	22 (57.9)
>3 to 4 liters	154 (26.6)	140 (25.9)	25 (20.8)	16 (20.5)	36 (27.3)	25 (28.1)	38 (31.1)	14 (36.8)
>4 liters	28 (4.8)	26 (4.8)	1 (0.8)	3 (3.8)	9 (6.8)	3 (3.4)	10 (8.2)	2 (5.3)

*Number of dairy cows.

#### Feeding management and housing conditions in the first 2 weeks of life

During the first 2 weeks of life, preweaned calves in all herds were predominantly fed whole milk (59.7%), followed by acidified whole milk (15.8%), and milk replacer (15.6%, Table 4). The most common maximum volume offered to calves at that age was 6 l per day (44.5%, Table 4). On one quarter of the farms (25.3%), more than 6–8 l per day were fed per calf. Overall, 15% of the farms offered a volume of more than 16 l per day (Table 4). The majority of farms fed the calves twice daily (23). In the first 2 weeks of life, on conventional farms, it was common that calves were kept in single housing (92.9%, Table 4). Every fifth organic farm (23.8%, Table 4) housed calves in groups.

**Table 4:** Feeding management and housing conditions of preweaned dairy calves in the first 2 weeks of life on 730 German dairies stratified by herd size and farm type.

		***N*** **(%)**						
		**Conventional**						
	**Total (*n* = 730)**	**Overall (*n* = 666)**	1–40*(*n* = 130)	41–60*(*n* = 99)	61–120*(*n* = 180)	121–240*(*n* = 119)	>241*(*n* = 138)	Organic (*n* = 64*)
**Feeding management**								
Whole milk	436 (59.7)	385 (57.9)	100 (76.9)	57 (57.6)	98 (54.7)	66 (55.5)	64 (46.4)	50 (78.1)
Milk replacer	114 (15.6)	114 (17.1)	9 (6.9)	14 (14.1)	39 (21.8)	24 (20.2)	28 (20.3)	0 (0.0)
Acidified whole milk	115 (15.8)	108 (6.2)	13 (9.9)	16 (16.2)	23 (12.8)	16 (13.4)	40 (29.0)	7 (10.9)
Others^+^	63 (8.6)	58 (8.7)	8 (6.1)	12 (12.1)	19 (10.6)	13 (10.9)	6 (4.3)	5 (7.8)
**Offered volume of liquid diet per day**								
< 6 l	323 (44.5)	299 (45.0)	67 (51.5)	49 (50.0)	88 (48.9)	51 (42.9)	44 (32.1)	23 (37.7)
>6–8 l	184 (25.3)	169 (25.5)	28 (21.4)	29 (29.6)	39 (21.7)	31 (26.1)	42 (30.7)	15 (24.6)
>8–10 l	82 (11.3)	73 (11.0)	17 (13.0)	7 (7.1)	15 (8.3)	15 (12.6)	19 (13.9)	9 (14.8)
>10–16 l	28 (3.9)	26 (3.9)	5 (3.8)	2 (2.0)	13 (7.2)	4 (3.4)	2 (1.5)	2 (3.3)
>16 l	109 (15.0)	97 (14.6)	13 (9.9)	11 (11.2)	25 (13.9)	18 (15.1)	30 (21.9)	12 (19.7)
**Housing**								
Single	678 (92.9)	629 (94.4)	123 (94.6)	96 (97.0)	170 (94.4)	111 (93.3)	129 (93.5)	48 (76.2)
Group	52 (7.1)	37 (5.6)	7 (5.3)	3 (3.0)	10 (5.6)	8 (6.7)	9 (6.5)	15 (23.8)

*Number of dairy cows.

^+^Mix of whole milk and milk replacer, yogurt, 1st week whole milk, 2nd week milk replacer.

#### Feeding management and housing conditions from the 3rd week of life

From the 3rd week of life, milk replacer (49.0%) was the main feed component on conventional farms, followed by whole milk (32.1%), other liquid diets, such as a mix of milk replacer and whole milk or yogurt (10.1%), and acidified whole milk (7.5%, Table 5). Organic farms did not offer milk replacer to their calves. More than 6–8 l per day was the most common volume of liquid diets (36.5%), followed by more than 8–10 l per day (27.1%, Table 5). In this age group, more than 16 l per day were offered less frequently (7.4%, Table 5). The majority of farms fed the calves twice daily (23). From the third week of life, it was more common for all herds to keep preweaned calves in group housing (81.9%, Table 5).

**Table 5:** Feeding management and housing conditions of preweaned dairy calves from the 3rd week of life on 730 German dairies stratified by herd size and farm type.

		***N*** **(%)**						
		**Conventional**						
	**Total (*n* = 730)**	**Overall (*n* = 666)**	1–40*(*n* = 131)	41–60*(*n* = 99)	61–120*(*n* = 180)	121–240*(*n* = 119)	>241*(*n* = 138)	Organic(*n* = 64*)
**Feeding management**								
Whole milk	235 (32.1)	185 (27.8)	66 (50.8)	31(31.3)	48 (26.7)	25 (21.1)	15 (10.9)	49 (76.6)
Milk replacer	358 (49.0)	358 (53.8)	38 (29.2)	41(41.4)	94 (52.2)	80 (67.2)	105 (76.1)	0 (0.0)
Acidified whole milk	55 (7.5)	47 (7.1)	8 (6.2)	11 (11.1)	14 (7.8)	2 (1.7)	12 (8.7)	8 (12.5)
Others^+^	74 (10.1)	69 (10.4)	17 (13.0)	16 (16.2)	21 (11.7)	10 (8.4)	5 (3.6)	5 (7.8)
**Volume of liquid diet per day**								
0–6 l	122 (17.1)	115 (17.6)	17 (13.1)	14 (14.4)	44 (25.0)	23 (19.8)	17 (12.5)	7 (11.7)
>6–8 l	261 (36.5)	243 (37.2)	40 (31.0)	41 (35.3)	64 (36.4)	41 (42.3)	57 (41.9)	18 (30.0)
>8–10 l	194 (27.1)	179 (27.4)	38 (29.5)	24 (24.7)	44 (25.0)	37 (31.9)	36 (26.5)	14 (23.3)
>10–16 l	85 (11.9)	74 (11.3)	29 (22.5)	13 (13.4)	17 (9.7)	7 (6.0)	8 (5.9)	11 (18.3)
>16 l	53 (7.4)	43 (6.6)	5 (3.9)	5 (5.2)	7 (4.0)	8 (6.9)	18 (13.2)	10 (16.7)
**Housing**								
Single	125 (17.1)	115 (17.3)	47 (36.2)	27 (27.3)	25 (14.0)	9 (7.6)	7 (5.1)	10 (15.6)
Group	597 (81.9)	542 (81.6)	81 (62.3)	72 (72.7)	152 (84.9)	108 (90.8)	140 (94.2)	54 (84.4)

*Number of dairy cows.

^+^Mix of whole milk and milk replacer, yogurt.

#### Weaning

Overall, the median age at weaning was 11 (IQR: 10–12) weeks of life. The weaning age varied slightly depending on herd size (Table 1). Farms with 41–60 dairy cows offered liquid feeding for a longer period of time (12 weeks) compared to those with more than 241 dairy cows (10 weeks). On organic farms, the calves were completely weaned at a median age of 12 (IQR 12–14) weeks (Table 1).

#### Random sample of calves for clinical examination

Up to 73 preweaned calves, all calves on farm were clinical examined. When this number was exceeded, a random sampling was taken [details are published elsewhere (1)]. Nevertheless, in a few cases more than 73 calves were examined by mistake. This resulted in a true maximum of 75 calves being examined per farm. The sample calculation was performed with a prevalence of 40% at a confidence level of 95% with a power of 80% and a precision of ± 5% being expected (1, 25).

#### Clinical examination and definition of disorders

Overall, a clinical examination by trained veterinarians was performed on a median of 12 (IQR: 6–25) preweaned dairy calves. The number of calves varied according to herd size, with a median of 5 to a median of 41 preweaned calves being examined per farm (Table 1). The clinical examination included auscultation of the lungs, palpation of the external umbilical structures, visual examination of the limbs at rest and in motion, taking the rectal temperature, and visual assessment of the fecal consistency [for a detailed description of the clinical examination, see (1)]. All findings were recorded on a data sheet using a scoring system. Assigning clinical signs to different disorders was based on predefined criteria for pathognomonic symptoms (case definition shown in Table 6). The following disorders were addressed: diarrhea (D), omphalitis (O), abnormal weight bearing (AWB), and respiratory disease (RD).

**Table 6:** Case definition of disorders based on characteristic clinical signs detected in the clinical examination.

**Clinical examination**	Characteristic clinical sign*		**Disorder**
Visual examination of the limbs at rest and in movement	Unequal load of at least one limb **OR** congenital contracture of the flexor tendons	+/− Other findings	Abnormal weight bearing **(AWB)**
Auscultation of the lungs	Increased, louder breathing sounds	+ Fever - Liquid or soft feces +/− Other findings	
	Reduced, low to complete absence of normal breathing sounds (“silent lung”)	+/− Other findings	
	Additional sounds besides normal breathing sounds including crackles or wheezes	+/− Other findings	Respiratory disease **(RD)**
	Reinforcement of the tracheobronchial breathing; breathing sounds that in healthy calves are only heard over the large airways (e. g. the trachea) can be heard over the chest wall	+/− Other findings	
Palpation of external umbilical structures	Inflammatory navel abnormalities: thickening and/or swelling and/or pain and/or heat, excluded uncomplicated umbilical hernia	+/− Other findings	Omphalitis **(O)**
Determination of fecal consistency (directly from rectum)	Feces watery or soupy (runs through fingers)	+/− Other findings	Diarrhea **(D)**
Measurement of transrectal body temperature	>39.5°C^**a**^	Evaluation only in combination with	
	< 38.0°C^**b**^	other clinical signs	

*The presence of the clinical sign is sufficient for diagnosis; +/− clinical sign may be present but does not have to be present; − clinical sign must not be present for diagnosis; + clinical sign must be present for diagnosis.

^**a**^Defined as fever.

^**b**^Defined as low body temperature.

Calves showing characteristic clinical signs of more than one disorder (e.g., thickening of the umbilical structures, and liquid or soft feces) were classified as multimorbid (multimorbidity, M).

### Statistical analysis

All statistical analyses were performed using R version 4.1.3 (R Foundation for Statistical Computing, Vienna). Descriptive tables were created using the tableone R package [version 0.13.0, (27)]. The prevalence of disorders was calculated as the percentage of the number of examined sick calves to the number of all examined calves on farm. Reference values for farm-level prevalences were calculated as 10, 25, 50 (median), 75, and 90% quantiles. Two repeated ANOVA measurements were taken to calculate the *p*-values for the effect of farm size and organic management on the prevalence. For both models, a random effect for region was included to account for clustering. Due to large differences in farm size between organic and conventionally managed farms, the organic management model was additionally adjusted for farm size. A *p*-value ≤ 0.05 was considered significant.

To account for the higher variability in prevalence on smaller farms, we used funnel plots. These show the confidence interval around the average prevalence for a given number of observations (i.e., number of calves) on the farm (28). Since confidence intervals are wider on farms with a lower number of observations (i.e., calves), this addresses the inherently higher variation in the measured prevalence in smaller farms. The confidence intervals were calculated using the modified Jeffreys method, which are equally tailed and provide better coverage close to 0 and 100% (29). Confidence intervals were calculated for 95 and 99.9% confidence levels and both upper and lower limits were plotted.

### Development of the calf health calculator

To calculate reference values for the prevalence at 10, 25, 50, 75, and 90% quantiles based on the number of calves, farm type (organic, conventional), and season, quantile non-parametric additive models were used [R package qgam version 1.3.4, (30)]. The seasonal effect was modeled as a circular spline based on the day of year. The effect of the number of calves was modeled as a restricted cubic spline to account for the higher variability in the upper quantiles (75 and 90%) of prevalence for smaller sample sizes (see funnel plot). The estimated model formed the basis for a spreadsheet using Microsoft^®^ Excel^®^ to allow stand-alone and offline on-farm usage of the farm-specific reference data.

## Results

### Overall prevalence of disorders on herd level independent of farm type (organic and conventional farms)

The overall prevalence of disorders presented in Table 7 can be used as general reference values in the daily counseling practice. The estimation of the prevalence of the following disorders was conducted for 730 dairies with a total of 13,658 preweaned dairy calves. At herd level, omphalitis (O, median 15.9%) was the most common disorder, followed by diarrhea (D, 15.4%) and respiratory disease (RD, 2.9%). Abnormal weight bearing (AWB) was rarely detected (median, 0.0%). Calves with symptoms of more than one disorder at the same time (multimorbidity, M) were observed with a median herd level prevalence of 2.3%. In these multimorbid calves, disease combinations of O, D, and RD occurred most frequently.

**Table 7:** Herd prevalence of disorders in 13,658 preweaned calves on 730 German dairies.

**Disorder**	**Q_0.1_**	**Q_0.25_**	**Median**	**Q_0.75_**	**Q_0.9_**	**Mean**
Respiratory disease (RD)	0.0	0.0	**2.9**	12.2	21.2	7.8
Diarrhea (D)	0.0	0.0	**15.4**	26.2	37.6	17.1
Abnormal weight bearing (AWB)	0.0	0.0	**0.0**	0.0	1.5	1.0
Omphalitis (O)	0.0	4.3	**15.9**	30.2	50.0	20.6
Multimorbidity (M)*	0.0	0.0	**2.3**	10.0	17.6	6.4
M_RD^*a^	0.0	0.0	**0.0**	2.6	8.3	2.4
M_D^*a^	0.0	0.0	**0.0**	7.7	14.3	4.8
M_AWB^*a^	0.0	0.0	**0.0**	0.0	0.0	0.4
M_O^*a^	0.0	0.0	**0.0**	8.3	16.7	5.4

Q_0.1_: 10%-quantile; Q_0.25_: 25%-quantile; Q_0.75_: 75%-quantile; Q_0.9_: 90%-quantile.

*Calves showing characteristic clinical signs of more than one disorder at the same time.

^a^Each subset of superordinate group Multimorbidity (M) with the occurrence of the following disorder combinations: RD, D, AWB, and O. Bold indicates the median values.

### Prevalence of disorders on conventional farms stratified by the number of dairy cows

There was a noticeable trend that with an increasing number of dairy cows in a herd, the prevalence of diarrhea (D), omphalitis (O), and multimorbidity (M) also increased. This concerned especially farms with more than 41 dairy cows. On farms with more than 61 dairy cows, the prevalence level of respiratory disease (RD) partly decreased with increasing herd size. The *p*-values for the effect of farm size were calculated as follows: for D (*p* < 0.001), RD (*p* = 0.008), AWB (*p* = 0.651), O (*p* = 0.135), and M (*p* = 0.098). Due to differences between herds in the prevalence of disorders, it was useful to compare farms with similar numbers of dairy cows. The differences in prevalence due to farm size are presented in Tables 8A–E.

**Table 8A:** Herd prevalence of disorders in preweaned dairy calves on 130 farms with 1 to 40 dairy cows^*^.

**Disorder**	**Q_0.1_**	**Q_0.25_**	**Median**	**Q_0.75_**	**Q_0.9_**	**Mean**
Respiratory disease	0.0	0.0	**0.0**	10.8	25.0	6.7
Diarrhea	0.0	0.0	**0.0**	24.3	40.0	12.3
Abnormal weight bearing	0.0	0.0	**0.0**	0.0	0.0	1.4
Omphalitis	0.0	0.0	**0.0**	20.0	50.0	13.5
Multimorbidity	0.0	0.0	**0.0**	0.0	14.3	4.0

*At the day of farm visit with a median of 5 (IQR: 2.5–7.0), calves underwent clinical examination.

Q_0.1_: 10%-quantile; Q_0.25_: 25%-quantile; Q_0.75_: 75%-quantile; Q_0.9_: 90%-quantile. Bold indicates the median values.

**Table 8B:** Herd prevalence of disorders in pre-weaned dairy calves on 99 farms with 41–60 dairy cows^*^.

**Disorder**	**Q_0.1_**	**Q_0.25_**	**Median**	**Q_0.75_**	**Q_0.9_**	**Mean**
Respiratory disease	0.0	0.0	**0.0**	11.1	25.6	9.1
Diarrhea	0.0	0.0	**15.4**	33.3	41.8	18.7
Abnormal weight bearing	0.0	0.0	**0.0**	0.0	1.1	0.9
Omphalitis	0.0	0.0	**12.5**	33.3	50.0	19.2
Multimorbidity	0.0	0.0	**0.0**	11.4	20.0	6.5

*At the day of farm visit with a median of 9 (IQR: 6.0–12.0), calves underwent clinical examination.

Q_0, 1_: 10%-quantile; Q_0, 25_: 25%-quantile; Q_0, 75_: 75%-quantile; Q_0, 9_: 90%-quantile. Bold indicates the median values.

**Table 8C:** Herd prevalence of disorders in pre-weaned dairy calves on 180 farms with 61–120 dairy cows^*^.

**Disorder**	**Q_0.1_**	**Q_0.25_**	**Median**	**Q_0.75_**	**Q_0.9_**	**Mean**
Respiratory disease	0.0	0.0	**5.7**	14.3	23.9	9.0
Diarrhea	0.0	0.0	**14.9**	25.0	35.2	16.4
Abnormal weight bearing (AWB)	0.0	0.0	**0.0**	0.0	0.0	0.9
Omphalitis (O)	0.0	9.1	**21.2**	40.3	60.0	26.1
Multimorbidity	0.0	0.0	**0.0**	12.5	19.0	7.3

*At the day of farm visit with a median of 12.0 (IQR: 8.0–17.0), calves underwent clinical examination.

Q_0.1_: 10%-quantile; Q_0.25_: 25%-quantile; Q_0.75_: 75%-quantile; Q_0.9_: 90%-quantile. Bold indicates the median values.

**Table 8D:** Herd prevalence of disorders in pre-weaned dairy calves on 119 farms with 121–240 dairy cows^*^.

**Disorder**	**Q_0.1_**	**Q_0.25_**	**Median**	**Q_0.75_**	**Q_0.9_**	**Mean**
Respiratory disease	0.0	0.0	**5.6**	12.5	19.1	8.2
Diarrhea (D)	0.0	7.9	**15.4**	22.9	36.6	17.3
Abnormal weight bearing (AWB)	0.0	0.0	**0.0**	0.0	3.4	1.3
Omphalitis (O)	7.1	12.5	**18.1**	31.6	52.6	24.0
Multimorbidity	0.0	0.0	**5.3**	10.0	17.4	6.8

*At the day of farm visit with a median of 21 (IQR: 16.0–29.0), calves underwent clinical examination.

Q_0.1_: 10%-quantile; Q_0.25_: 25%-quantile; Q_0.75_: 75%-quantile; Q_0.9_: 90%-quantile. Bold indicates the median values.

**Table 8E:** Herd prevalence of disorders in pre-weaned dairy calves on 138 farms with more than 241 dairy cows^*^.

**Disorder**	**Q_0.1_**	**Q_0.25_**	**Median**	**Q_0.75_**	**Q_0.9_**	**Mean**
Respiratory disease	0.0	2.6	**5.3**	10.2	15.4	7.0
Diarrhea (D)	8.6	14.9	**21.0**	29.2	36.0	22.7
Abnormal weight bearing (AWB)	0.0	0.0	**0.0**	0.0	2.5	0.5
Omphalitis (O)	9.5	12.9	**19.5**	26.8	35.0	20.8
Multimorbidity	0.9	2.9	**5.8**	10.5	16.2	7.6

*At the day of farm visit with a median of 41 (IQR: 37.0–71.0), calves underwent clinical examination.

Q_0.1_: 10%-quantile; Q_0.25_: 25%-quantile; Q_0.75_: 75%-quantile; Q_0.9_: 90%-quantile. Bold indicates the median values.

#### A total of 1–40 dairy cows

On farms with 1 to 40 dairy cows (*n* = 130) a minimum of one to a maximum of 19 preweaned calves were examined (median: 5; IQR 3–7). Clinical examination was performed for all preweaned calves (median 100%). On at least 50% of the farms, no calves with disorders were detected (median, 0.0%). In the 75%-quantile of farms, every 4th calf had D (24.3%), every 5th calf had O (20.0%), and every 10th calf had RD (10.8%, Table 8A).

#### A total of 41–60 dairy cows

On farms with 41 to 60 dairy cows (*n* = 109) all preweaned calves on farm were examined (median 100%; IQR: 86–100%). Clinical examination was conducted for at least one calf to a maximum of 28 calves (median: 9; IQR: 6–12). When considering the median, D was the most common disorder (15.4%), followed by O (12.5%). On at least 50% of the farms, no calves with RD, AWB, and M were observed (median 0.0%). In the 75%-quantile of farms, at least one third of the examined calves had O (33.3%) or D (33.3%) and at least more than 1 of 10 calves suffered from RD (11.1%) or M (11.4%, Table 8B).

#### A total of 61–120 dairy cows

At least 2 to a maximum of 46 preweaned calves per farm were examined (median: 12; IQR: 8–17) in a herd size with 61–120 dairy cows (*n* = 180). This corresponds to almost all presented preweaned calves on farm (median 100%; IQR: 86–100%). In this herd size group, O was the most common disorder with a median herd level prevalence of 21.2%, followed by D (14.9%), and RD (5.7%). On at least 50% of the farms, no calves with more than one disorder at the same time (multimorbidity) were detected (median 0.0%). In the 25%-quantile of the farms, almost 1 of 10 calves had an omphalitis (9.1%, Table 8C).

#### A total of 121–240 dairy cows

In a herd size between 121 and 240 dairy cows (*n* = 119) at least 3 to a maximum of 63 preweaned calves were examined (median of 21; IQR: 16–29). Of the total number of calves on farm, on median 94% (IQR: 87–100%) of the calves were examined. When considering the median, O was the most common diagnosis (18.1%), followed by D (15.4%) and RD (5.6%). In the 25%-quantile of farms, D and O were detected with a prevalence of 7.9 and 12.5%, respectively. On the 10% of farms with the lowest prevalence, O was found in 7.1% of the examined calves (Table 8D).

#### A total of 241 and more dairy cows

On farms with more than 241 (Max: 2,821) dairy cows (*n* = 138) at least 5 to a maximum of 75 preweaned calves were examined (median 41; IQR: 37–71). Clinical examination was carried out for 79% (IQR: 64–89%) of the total number of calves on farm. Diarrhea (21.0%) was the most common diagnosis, followed by O (19.5%), and RD (5.3%). In the 25%-quantile of farms, D, O, and RD were detected with a prevalence of 14.9, 12.9, and 2.6%, respectively. Even on the 10% of farms with the lowest prevalence, calves with D (8.6 %), O (9.5%), and M (0.9%) were found (Table 8E).

### Prevalence of disorders in calves on organic farms

Table 9 assesses the health status of the preweaned calves on organic farms. The organic farms (*n* = 64) enrolled in this study had a median of 42 (IQR: 27–80; Min: 1; Max: 297) dairy cows. At the day of the farm visit, at least one to a maximum of 33 calves were examined (median: 6; IQR: 3–11). Considering the median (IQR: 89–100%), an examination of all preweaned calves on farm was conducted. The most common calf disorders observed on organic farms were diarrhea (D, 8.7%) and omphalitis (O, 8.5%). On at least 50% of the organic farms, no calves with respiratory disease (RD), abnormal weight bearing (AWB) and multimorbidity (M) were observed. In the 75%-quantile of farms, every 4th calf had D (25.0%) and every 5th calf had O (20.8%). Respiratory disease (4.7%) and M (4.1%) were detected to a lesser extent (Table 9). There was a noticeable tendency for organic farms to have a lower prevalence of disorders than similar sized conventional farms. The farm-size adjusted *p*-values of this effect were calculated for the following disorders: D (*p* = 0.092), RD (*p* = 0.082), AWB (*p* = 0.127), O (*p* = 0.295), and M (*p* = 0.441). Although a difference in the prevalence of disorders was observed between organic and similar sized conventional farms, however, this was not statistically significant.

**Table 9:** Herd prevalence of disorders on herd level on 64 organic farms^*^.

**Disorder**	**Q_0.1_**	**Q_0.25_**	**Median**	**Q_0.75_**	**Q_0.9_**	**Mean**
Respiratory disease	0.0	0.0	**0.0**	4.7	19.0	5.5
Diarrhea (D)	0.0	0.0	**8.7**	25.0	33.3	14.2
Abnormal weight bearing (AWB)	0.0	0.0	**0.0**	0.0	0.0	0.2
Omphalitis (O)	0.0	0.0	**8.5**	20.8	47.0	14.7
Multimorbidity	0.0	0.0	**0.0**	4.1	14.3	4.9

*At the day of farm visit with a median of 6 (IQR: 3.0–11.0), calves underwent clinical examination.

Q_0.1_: 10%-quantile; Q_0.25_: 25%-quantile; Q_0.75_: 75%-quantile; Q_0.9_: 90%-quantile. Bold indicates the median values.

### Evaluation prevalence adjusted for the number of examined calves (funnel plots)

**Figure 1:**
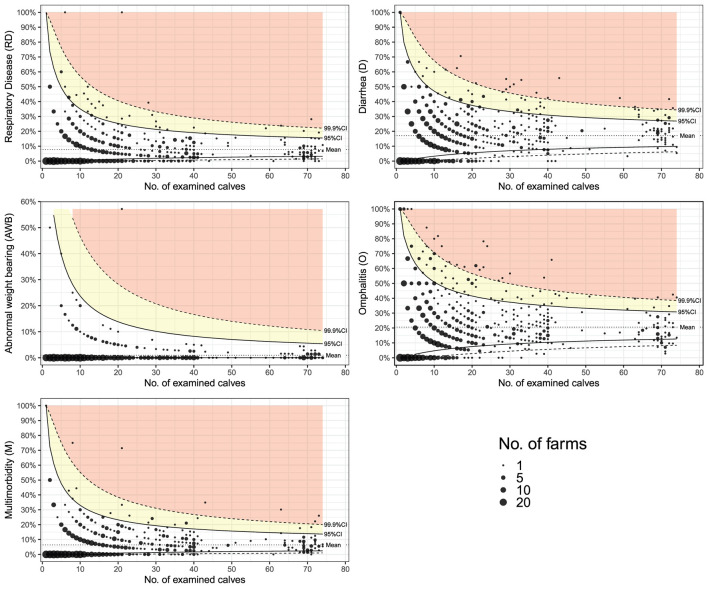
Prevalence of disorders stratified by number of examined preweaned dairy calves on the farm. The dotted line marks the mean, the solid line marks the upper and lower 95% CI, and the dashed line marks the upper and lower 99.9% CI. In cases of sporadic occurrence, not more than 1 in 20 farms should lie outside of the interval for 95% CI (yellow marked area) and not more than 1 in 1,000 for 99.9% CI (red marked area). The size of the spots represents the number of farms with the similar prevalence of disorders and number of examined calves. Therefore, there are more farms included, as the size of the dot increases.

The funnel plots (Figure 1) visualize the distribution of the prevalences of disorders according to the number of calves on the farm. The number of farms can also be determined from the size of the dots in the diagram. For every possible number of examined calves (up to 75) on a farm, confidence intervals around the overall average prevalence were calculated. The confidence intervals were calculated for 95 and 99.9% confidence intervals. The lower the number of calves, the wider the confidence interval. The confidence intervals can be used to assess whether the disorders occur sporadically, are randomly distributed, or occur at an increased rate. In cases of sporadic occurrence, not more than 1 in 20 farms should lie outside of the interval for 95% CI (yellow marked area) and not more than 1 in 1,000 for 99.9% CI (red marked area). If more farms are outside of the CI, this can be an indication that major outbreaks are common for this particular disease. For the individual farm, this indicates a major outbreak is in progress. For example, it can be observed that diarrhea and omphalitis often lead to larger outbreaks (Figure 1). For abnormal weight bearing, there is no increased clustering within farms. Respiratory disease also shows a minor tendency for an increased frequency of occurrence, and multimorbidity results from a combination of all disorders.

### Calf health calculator

#### Prevalence depending on season, farm size, and farm type

**Figure 2:**
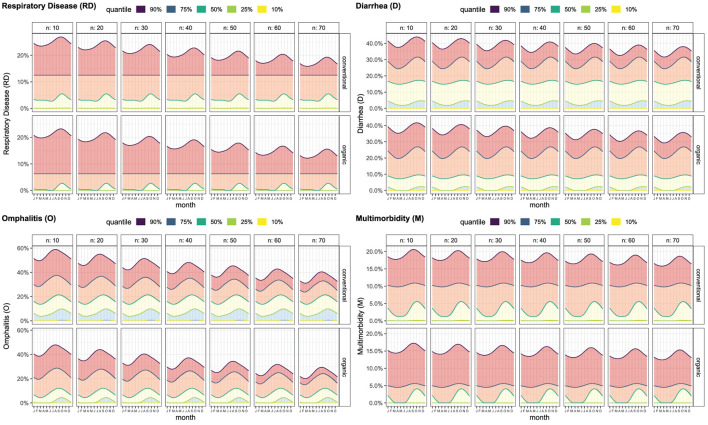
Prevalence of disorders based on number of preweaned dairy calves examined at the day of the farm visit for conventional and organic managed dairy farms depending on day of year/month. Quantile functions were estimated using a quantile non-parametric additive model (qgam) for 10, 25, 50, 75, and 90% quantiles. A continuous effect was estimated using restricted splines for number of calves and day of year (circular). Exemplary values are shown for the number of calves (*n* = 10, *n* = 20, *n* = 30, etc.). The farm type (organic, conventional) effect was estimated as a factor. These functions form the basis for the calf health calculator.

Figure 2 shows the estimated quantile functions, which are the basis for the calf health calculator. The seasonal effect was modeled as a circular spline (i.e., after December comes January) based on the day of the year. The number of calves was estimated as a restricted cubic spline and estimated values are given exemplarily (*n* = 10, *n* = 20, *n*= 30, etc.) for conventional and organic farms. The level of prevalence in the 90%-quantile decreased with increasing number of examined calves on the farm. The levels of the other quantiles (Q_0.1_, Q_0.25_, median, Q_0.75_) were not affected by the number of examined calves on the farm. Respiratory diseases, D, and M occurred more frequently in the fall. Omphalitis was most common in the summer months. At the individual animal level, it was already determined that no seasonal effect can be represented for abnormal weight bearing (1). Therefore, in the present study, the prevalence of abnormal weight bearing by season is not illustrated.

#### Calf health calculator in excel

On the basis of the results obtained in the present study, a digital calculator was developed that allows for classification of the health status of preweaned dairy calves as determined on a farm visit through real-time access by comparison with the underlying study population. Reference values for the disease prevalence for respiratory disease (RD), diarrhea (D), omphalitis (O), and multimorbidity (M) were included. A model was fitted to account for seasonal effects, farm type (organic, conventional) and number of examined calves. As reference values, the 10, 25, 50 (median), 75, and 90% quantiles of the observed data were used. The estimated reference values were entered into a spreadsheet for on-farm use. The calf health calculator estimates the farm-specific prevalence and benchmarks the results using the reference values based on the underlying study population automatically. The calculator is available as a stand-alone tool for Microsoft^®^ Excel^®^ (https://doi.org/10.6084/m9.figshare.c.6172753).

## Discussion

The present study for the first time provides a deep insight into the health status of preweaned dairy calves on herd-level in three different regions of Germany on farms that differ in herd size, management, and farm type (conventional, organic). Data were obtained from databases of the testing associations affiliated with the farms and collected during farm visits on a single occasion by interview, visual observations, and clinical examinations of individual calves. The results proved to be suitable to establish benchmarks that allow for comparisons of the health status of preweaned dairy calves on farms by herd size, farm type, and month of the year. A digital tool (“calf health calculator”) was developed that enables farmers, veterinarians and other advisory persons, to classify the health-status of preweaned calves in real-time subsequent to on-farm data acquisition on a farm visit.

### Pattern validity of the data set and qualification as reference values

To our knowledge, the “PraeRi” (1, 25) study is the first cross-sectional study presenting such an extensive and diverse dataset including the description of the prevalence of disorders of preweaned dairy calves on German dairies. The final data set included 730 dairy farms with a total of 13,658 preweaned dairy calves. The surveyed farms were located in three different regions of Germany with intensive dairy farming (31) and exhibited marked differences in herd size, structure and management practices (1). These different farms represent a wide variety of production characteristics and thus allow a wide variety of different effects of potential risk factors under field conditions. The farm visits took place continuously in a 3-year period (December 2016 to July 2019). The farms were randomly selected, and regular monitoring of the selection process ensured a high level of certainty in the random sample of farms. The clinical examination of the preweaned calves was performed by 21 trained veterinarians employed explicitly for this study. Trainings for this purpose was conducted and standard operation procedures were developed. In addition, regular meetings and interobserver reliability tests were performed to ensure a high reliability of the recorded data (1). Thus, the estimated prevalences are suitable as reference values for benchmarking dairy farms with a wide range of different herd size, management structures, and geographic location. Due to the diversity of German dairy farming, these data are useful for comparing similarly structured farms in other regions and countries as well.

### Alignment of the estimated herd level prevalence with previous studies

In the current study, the overall median herd level prevalence for diarrhea (D, 15.4%) was slightly higher than reported from Chilian dairy farms (15). However, the authors suggest that the mean herd level prevalence of 12.7% may be underestimated due to the small number of examined calves and the fact that the diagnosis was only made by visual inspection (15). In a Canadian study (32), the median within-pen prevalence for D was 17.0% (IQR: 7.0–37.0%). In 19 commercial dairies in Minnesota and Ontario a median herd level incidence risk of 10.5% for D was determined (12). The estimated median herd level prevalence for respiratory disease (RD, 2.9%) corresponds to results from a Norwegian cross-sectional study (33). Other authors, reported noticeable higher prevalence for RD. In a Chilian study, a median herd level prevalence of 17.7% for RD was detected (15) and in an US study 12.0% of the preweaned heifers were affected by RD (34).

### Prevalence of omphalitis

The results of this study indicate that omphalitis (O) accounted for the largest proportion of disorders (15.9%) at herd level. The literature is primarily focused on D and RD as the most common disorders in preweaned dairy calves (35–37). In contrast, there is only scarce literature on the prevalence and kind of O on dairy farms primarily focusing on male calves by observations during a limited period of time and under poor conditions (38–41). The diagnosis of O cannot be made on the basis of visual observation alone but requires the collection of vital parameters and palpation of the umbilical region of the calf. Therefore, it is likely that the diagnosis O is missed when monitoring calf health on farms. Analysis of the data at individual animal level demonstrates that O is frequently associated with other disorders such as D, RD, or AWB (1). In addition, omphalitis should also be considered as a possible cause of growth retardation (39), an increased susceptibility to other diseases and mortality (41) in preweaned dairy calves. Our findings show that farmers and veterinarians should devote special attention to the umbilicus at parturition and in the 1st days of life of the neonate. In addition, umbilical disorders should find more consideration in future research.

### Prevalence of disorders stratified by herd size

The results of the present study demonstrate clear associations between the herd size and the prevalence of disorders in dairy calves. From the literature, it is well-known that herd size has an effect on the prevalence of calf disorders (42–45). For the effect of farm size, a statistically significant difference for diarrhea and respiratory disease was found. An effect of farm size on omphalitis, abnormal weight bearing, and multimorbidity could also be observed, but this was not statistically significant. Herd size is usually known and therefore enables a comparison between similarly structured farms. Due to the diversity of dairy farming in Germany, the findings of this study can be used for comparison of similarly structured farms of other regions and countries. The proportion of calves on dairy farms affected by the disorders addressed in the present study increased with herd size. On smaller farms, fewer preweaned calves are kept, thus probably reducing the risk of infection, and leaving more time for individual care of calves. However, it should be noted that an effect of herd size could not be found for all studied diseases. Furthermore, as the number of calves examined on smaller farms was lower, the recorded diseases have a more pronounced effect on the herd level prevalence of the farm. Nevertheless, the authors assume as does Kaske (46) that in cases of high prevalence of disorders, there may be deficits in management and hygiene on the farm. Therefore, herd size should be taken into account when including disease prevalence in on-farm benchmarking.

### Herd size and omphalitis

The results of this study revealed that with increasing herd size the prevalence of omphalitis (O) increased as well. Omphalitis results from mixed bacterial infections of the umbilical structures (47). From the site of infection, bacteria can spread into single joints or even cause fatal systemic infections (48). It is likely that the higher number of calvings on large dairy farms increase the infection pressure, reduce the time spent on navel disinfection in individual calves or increase the occurrence of navel sucking in larger groups of calves of the same age (49). To prevent O, adequate hygiene in the maternity pen, quick removal of the newborn calves from the maternity pen as well as quick supply of larger volumes of high-quality colostrum (50, 51) are considered crucial. Disinfection of the umbilical structures after birth can also reduce the risk of infection (52).

### Herd size and respiratory disease

In farms with a herd size of < 60 dairy cows, when considering the median, no calves with respiratory diseases (RD) were detected. The highest prevalence of RD was observed for herd sizes of 61–120 dairy cows (5.7%). With increasing numbers of cows, the prevalence of RD slightly decreased. Housing conditions for the preweaned calves have a great impact on the occurrence of RD. On smaller farms, calves were more often kept in smaller groups ( ≤ 7 calves), which could have reduced the occurrence of RD (37, 53). In Germany, on farms with up to 120 dairy cows, it is more common to use old buildings for calf rearing and, in some cases, prophylactic measures are not implemented (26). In unventilated barns, the climatic conditions are often poor. Effects of sudden changes in ambient temperature and humidity, exposure to dust and toxic gases as well as deficient biosecurity measures promote higher prevalences of RD (8, 14). Moreover, on larger farms, it is more established to use prophylactic measures such as a vaccination against RD compared with smaller farms (54). In a Norwegian study it was observed that in larger herds (> 50 dairy cows), the number of animals susceptible to infection was also higher, which can lead to more infections during an outbreak (43). This also promotes the possibility of pathogens circulating within a herd over a longer period and thus can cause infections again and again (55).

### Herd size and diarrhea

The highest median herd level prevalence for diarrhea (21.0%) was recorded for farms with more than 240 dairy cows. In this herd size, the prevalence for diarrhea was also highest in the top 10- and 25%-quantile of the farms. Diarrhea is multifactorial by origin including infectious and non-infectious factors. The most common pathogens causing diarrhea in neonatal calves are enterotoxigenic *E. coli* (ETEC), rota- and coronaviruses, and cryptosporidia (56–58). The pathogens are present on every dairy farm (ubiquitous) and the infection happens *via* the environment (maternity pen, housing of calves, teat buckets, etc.) or by contact between calves. However, higher internal infection pressure on farms increases the risk of infection. Higher numbers of calvings and a high stocking density in combination with deficient hygienic conditions lead to an accumulation of pathogens in the animals‘ surroundings increasing the risk of infection (42). The latter is especially true for cryptosporidium that can survive for a long time in the environment (44).

### Herd size and multimorbidity

The proportion of multimorbid calves increased with increasing herd size. The occurrence of diseases, especially in the first 2 weeks of life, can promote the development of other diseases (12, 59). The previous disorders may cause immunosuppression and may result in a vulnerability to further diseases (60). Moreover, with increasing herd size the time for health monitoring of the individual calf will decrease (42). This can limit the timely detection and treatment of the disease which can lead to the manifestation of more than one disorder simultaneously.

### Calf rearing strategies on organic farms and prevalence of disorders

Due to the clear differences in structure and management of organic and conventional farms, a separate assessment of the health status of the preweaned calves is indicated. Organic farms tended to have fewer dairy cows (median 42) compared to conventional farms. A cross-sectional study in Michigan and Ohio, USA (54) also showed significant differences in management practices between organic and conventional farms. Similar to our observations on conventional farms, it was more common to hand feed the colostrum (304 of 448 farmers), whereas the majority of organic farmers (69 of 171) let the calves suck the dam for colostrum intake. In the same study (54), it was observed that conventional producers separated the calves from the dam earlier after birth compared to organic producers. There is already a study from the United States reporting parameters of the health status of preweaned dairy calves on organic farms. In this study, the incidences for D (44.4%) and RD (11.5%) were significantly higher than the prevalence reported in the current study. Possible reasons for these differences may be that the farms in the US study were not randomly selected, and that the disease recording was not done by veterinarians but by farm personnel (26). To our knowledge, ours is the first representative cross-sectional study reporting the prevalence of disorders in preweaned dairy calves on organic farms. In the current study, there is a noticeable tendency for organic farms to have a lower prevalence of disorders than similar sized conventional farms. However, this effect is not statistically significant. The impact of these or other unrecorded management factors, especially of organic farms on the prevalence of disorders in preweaned dairy calves needs to be clarified in further studies.

### Prevalence of disorders depending on season

In the fall and winter, respiratory diseases, diarrhea, and multimorbidity had the highest prevalence. Calves born in the fall had a 1.8- and 2.0-times higher risk of being treated for diarrhea compared to those born in the spring or summer (12). Possible causes of higher disease rates may be a lower colostrum quality in winter (61, 62) and a higher shedding of the pathogens (e.g., cryptosporidium) compared to the summer season (44). The lower temperature and the higher humidity in the fall and winter months provide better survival chances, for example the oocytes of cryptosporidium (63). In contrast, the highest prevalence of omphalitis was detected in the summer. A wide range of opportunistic bacteria are often involved in umbilical infections (46). With increasing temperature, the bacteria in the environment proliferate, which might increase the risk of infection. In the summer months, dust and flies can also act as predisposing factors (64).

### Hands-on applications and calf health calculator

The added variability due to a low number of observations is a major problem when comparing farm level prevalence on small farms with reference data. On farms with three calves, the prevalence can only be either 0% (0/3), 33% (1/3), 66% (2/3), or 100% (3/3). Thus, it is quite easy to observe a high prevalence due to random variation. To address this problem, in the present study, funnel plots were used for visualization (62). For every possible number of examined calves (up to 75) on a farm, confidence intervals around the overall average prevalence were calculated. The lower the number of calves, the wider the confidence interval (27). This addresses the problem of the higher variation due to a lower number of observations (i.e., calves). Funnel plots are helpful to easily identify sporadic occurrences or an outbreak of a disease (28). Based on the differences demonstrated for the prevalence of the individual disorders according to the number of calves, month of the farm visit, and the farm type (conventional, organic) these factors build the basis for the calf health calculator. As already discussed in the section on funnel plots (see above), the number of calves was added to account for the higher variability on farms with a lower number of calves, i.e., higher thresholds for small farms. Additionally, a continuous effect based on the day of year was estimated to account for seasonality in disease occurrence. Abnormal weight bearing was omitted because of the very low prevalence (1).

### Application in practice

The objective of this study was to transform the estimated prevalence for calf disorders based on a large and diverse data set into an applicable form for use in practice. In herd management of dairy farms, it is already common practice to use health data e.g., chewing activity, rumen fill, and fecal consistency for monitoring the health status of dairy cows. Farmers, herd managers, veterinarians and other advisors use tools based on these data to develop farm-specific concepts and management recommendations. In contrast, there are still no established uniform monitoring measures for calves. A Canadian study discovered that only one third of the veterinarians regularly asked about the health and performance of the calves on routine herd visits; as many as 13% of the surveyed veterinarians never asked about the calves (65). This is particularly problematic, as the consulting veterinarians play a key role in implementing changes in management practices to improve on-farm health (66) and are an important source of information about dairy herd health and management (67). Interviews with farmers revealed that they value communicating with the herd consulting veterinarian about calf health and development, and benchmarking can motivate them to make changes affecting calf management (22). In addition, farmers motivated by a trusted advisor were more likely to make changes in disease prevention management (68). Furthermore, benchmarking can help to reinforce the relationship between farmers and veterinarians (22).

The tools (table, diagrams, and digital calf health calculator) developed in this study will now be available to farmers, herd managers, veterinarians, and other advisors to help include internal or external calf health monitoring in their work routine. By documenting calf health on the farm using the calf health calculator, the authors hope to improve monitoring of calves on the farm, while detecting diseases more quickly and identifying potential problem areas. However, if the recording of the health status of the preweaned calves is carried out by non-trained personnel and in a less standardized way, as done in the present study, it is possible that measurement errors and deviations from the reported reference values may occur. Nevertheless, these differences in recording and classification are consistent within a person, so that this method is still suitable for assessing calf health on the farm. In addition, the documented data enables a permanent controlling and comparing within the farm as well as with other farms. Through the use of benchmarking, the authors expect that calves will become more visible in dairy farms as well as in consultancy practices, which may lead to sustained improvements in calf health.

Due to the size and diversity of the study population, these data allow farmers to compare themselves with similarly sized and structured farms. This high level of identification gives the data much greater credibility in consulting practice than reference values taken from farms that differ clearly in size, structure, and management. An update of the reference values applied in this study will not take place in the near future, because another study with such an extensive data set like the PraeRi study is not yet planned. In order to make these tools available for other study populations, the used code will be provided in the Supplementary material. Furthermore, a translation of the calf health calculator into other languages (currently German and English are available) is planned, as well as the development of a Libre Office version.

## Conclusion

At herd level, omphalitis (O) was the most detected disorder. This is particularly interesting because in the literature, diarrhea (D) and respiratory diseases (RD) are discussed as the main causes of calf disorders. Therefore, more attention should be paid to O in future studies and in the practice as well. Moreover, the current study demonstrated marked differences in the prevalence of disorders (D, RD, O, AWB, and M) between herds which partly could be explained by herd size, farm type (organic, conventional), and season. Thus, for a viable benchmarking, it is useful to take these factors into account. Overall, our results reveal that calf health should become a central issue for dairy farmers and in veterinary herd health consultancy. The benchmarks developed in this study should provide a practical tool for assessing on-farm calf health. Due to the extensive and diverse data set of the “PraeRi” study and the diversity of dairy cow farming in Germany, we assume that the results of this study can be transferred to other regions and countries as well.

## Data availability statement

The raw data supporting the conclusions of this article will be made available by the authors, without undue reservation.

## Ethics statement

Ethical review and approval was not required for the animal study because no painful interventions were made. This was in accordance with the local legislation and institutional requirements. Written informed consent was obtained from the owners for the participation of their animals in this study.

## Author contributions

LD, HA, ARB, LK, AnS, KB, PD, MK, PP, AlS, and SW visited the farms and collected the data. LD, AB, HA, and MH analyzed the data. AB, LD, HA, and MH developed the calf health calculator. LD, HA, and AB wrote the first draft of this work and discussed the results with MH. MH, KM, and GK-S acquired the funding for the realization of the project PraeRi. All authors were involved in the planning of the study, revised the article critically, and contributed substantial ideas. All authors contributed to the article and approved the submitted version.

## Funding

Funding of this project was provided by the Federal Ministry of Food and Agriculture and Federal Office for Agriculture and Food, grant numbers 2814HS006 (University of Veterinary Medicine Hannover, Foundation), 2814HS007 (Freie Universität Berlin), and 2814HS008 (Ludwig-Maximilian Universität Munich). This Open Access publication was funded by the Deutsche Forschungsgemeinschaft (DFG, German Research Foundation) - 491094227 “Open Access Publication Funding” and the University of Veterinary Medicine Hannover, Foundation.

## Conflict of interest

Author KB was employed by VetZ GmbH.

The remaining authors declare that the research was conducted in the absence of any commercial or financial relationships that could be construed as a potential conflict of interest.

## Publisher's note

All claims expressed in this article are solely those of the authors and do not necessarily represent those of their affiliated organizations, or those of the publisher, the editors and the reviewers. Any product that may be evaluated in this article, or claim that may be made by its manufacturer, is not guaranteed or endorsed by the publisher.

## Supplementary material

The Supplementary Material for this article can be found online at: https://www.frontiersin.org/articles/10.3389/fvets.2022.990798/full#supplementary-material; https://doi.org/10.6084/m9.figshare.c.6172753

## References

[1] Dachrodt L Arndt H Bartel A Kellermann LM Tautenhahn A Volkmann M. Prevalence of disorders in preweaned dairy calves from 731 dairies in Germany: a cross-sectional study. J Dairy Sci. (2021) 104:9037–51. 10.3168/jds.2021-20283 33985777

[2] Donovan GA Dohoo IR Montgomery DM Bennett FL. Calf and disease factors affecting growth in female holstein calves in Florida, USA. Prev Vet Med. (1998) 33:1–10. 10.1016/S0167-5877(97)00059-7 9500160

[3] Virtala AMK Mechor GD Gröhn YT Erb HN. The effect of calfhood diseases on growth of female dairy calves during the first 3 months of life in New York state. J Dairy Sci. (1996) 79:1040–9. 10.3168/jds.S0022-0302(96)76457-3 8827469 PMC7130866

[4] Heinrichs AJ Heinrichs BS. A prospective study of calf factors affecting first-lactation and lifetime milk production and age of cows when removed from the herd. J Dairy Sci. (2011) 94:336–41. 10.3168/jds.2010-3170 21183043

[5] Santman-Berends IMGA Schukken YH van Schaik G. Quantifying calf mortality on dairy farms: challenges and solutions. J Dairy Sci. (2019) 102:6404–17. 10.3168/jds.2019-16381 31056325

[6] Abuelo A. Symposium review: late-gestation maternal factors affecting the health and development of dairy calves. J Dairy Sci. (2020) 103:3882–93. 10.3168/jds.2019-17278 32037167

[7] Godden SM Lombard JE Woolums AR. Colostrum management for dairy calves. Vet Clin North Am Food Anim Pract. (2019) 35:535–56. 10.1016/j.cvfa.2019.07.005 31590901 PMC7125574

[8] Wójcik J Pilarczyk R Bilska A Weiher O Sanftleben P. Performance and health of group-housed calves kept in igloo calf hutches and calf barn. Pak Vet J. (2013) 33:175–8.

[9] Maccari P Wiedemann S Kunz HJ Piechotta M Sanftleben P Kaske M. Effects of two different rearing protocols for Holstein bull calves in the first 3 weeks of life on health status, metabolism and subsequent performance. J Anim Physiol Anim. (2015) 99:737–46. 10.1111/jpn.12241 25115790

[10] Kehoe SI Jayarao BM Heinrichs AJ. A survey of bovine colostrum composition and colostrum management practices on Pennsylvania dairy farms. J Dairy Sci. (2007) 90:4108–16. 10.3168/jds.2007-0040 17699028

[11] Marce C Guatteo R Bareille N Fourichon C. Dairy calf housing systems across Europe and risk for calf infectious diseases. Animal. (2010) 4:1588–96. 10.1017/S1751731110000650 22444707

[12] Windeyer MC Leslie KE Godden SM Hodgins DC Lissemore KD LeBlanc SJ. Factors associated with morbidity, mortality, and growth of dairy heifer calves up to 3 months of age. Prev Vet Med. (2014) 113:231–40. 10.1016/j.prevetmed.2013.10.019 24269039

[13] Karle BM Maier GU Love WJ Dubrovsky SA Williams DR Anderson RJ. Regional management practices and prevalence of bovine respiratory disease in California's preweaned dairy calves. J Dairy Sci. (2019) 102:7583–96. 10.3168/jds.2018-14775 30527977

[14] Lago A McGuirk SM Bennett TB Cook NB Nordlund KV. Calf respiratory disease and pen microenvironments in naturally ventilated calf barns in winter. J Dairy Sci. (2006) 89:4014–25. 10.3168/jds.S0022-0302(06)72445-6 16960078

[15] Calderon-Amor J Gallo C. Dairy calf welfare and factors associated with diarrhea and respiratory disease among Chilean dairy farms. Animals. (2020) 10:115. 10.3390/ani10071115 32610569 PMC7401522

[16] Valle PS Lien G Flaten O Koesling M Ebbesvik M. Herd health and health management in organic versus conventional dairy herds in Norway. Livest Sci. (2007) 112:123–32. 10.1016/j.livsci.2007.02.005

[17] Mee JF. Denormalizing poor dairy youngstock management: dealing with “farm-blindness”. J Anim Sci. (2020) 98:S140–s9. 10.1093/jas/skaa137 32810251 PMC7433914

[18] Egger-Danner C Köck A Fuchs K Grassauer B Fuerst-Waltl B Obritzhauser W. Use of benchmarking to monitor and analyze effects of herd size and herd milk yield on cattle health and welfare in Austrian dairy farms. J Dairy Sci. (2020) 103:7598–610. 10.3168/jds.2019-16745 32505408

[19] von Keyserlingk MAG Barrientos A Ito K Galo E Weary DM. Benchmarking cow comfort on North American freestall dairies: lameness, leg injuries, lying time, facility design, and management for high-producing Holstein dairy cows. J Dairy Sci. (2012) 95:7399–408. 10.3168/jds.2012-5807 23063152

[20] Fauteux V Roy J-P Scholl DT Bouchard É. Benchmarks for evaluation and comparison of udder health status using monthly individual somatic cell count. Can Vet J. (2014) 55:741–8. 25082989 PMC4095961

[21] Esslemont RJ Kossaibati MA. The use of databases to manage fertility. Anim Reprod Sci. (2000) 61:725–41. 10.1016/S0378-4320(00)00081-6 10844238

[22] Atkinson DJ von Keyserlingk MAG Weary DM. Benchmarking passive transfer of immunity and growth in dairy calves. J Dairy Sci. (2017) 100:3773–82. 10.3168/jds.2016-11800 28237586

[23] Sumner CL von Keyserlingk MAG Weary DM. How benchmarking motivates farmers to improve dairy calf management. J Dairy Sci. (2018) 101:3323–33. 10.3168/jds.2017-13596 29397181

[24] Sumner CL von Keyserlingk MAG Weary DM. How benchmarking promotes farmer and veterinarian cooperation to improve calf welfare. J Dairy Sci. (2020) 103:702–13. 10.3168/jds.2019-16338 31629510

[25] PraeRi. Final Report PraeRi, Animal Health, Hygiene and Biosecurity in German Dairy Cow Operations - A Prevalence Study. (2020). Available online at: https://ibei.tiho-hannover.de/praeri/pages/69 (accessed May 10, 2022).

[26] Haagen IW Hardie LC Heins BJ Dechow CD. Genetic parameters of calf morbidity and stayability for US organic Holstein calves. J Dairy Sci. (2021) 104:11770–8. 10.3168/jds.2021-20432 34419271

[27] Yoshida, K, Bartel, A,. Tableone: Create “Table 1” to Describe Baseline Characteristics With or Without Propensity Score Weights. R package version 012 0. Available online at: https://cran.r-project.org/web/packages/tableone/ (accessed May 20, 2020).

[28] Dover DC Schopflocher DP. Using funnel plots in public health surveillance. Popul Health Metrics. (2011) 9:58. 10.1186/1478-7954-9-58 22074228 PMC3254595

[29] Brown LD Cai TT DasGupta A. Interval estimation for a binomial proportion. Stat Sci. (2001) 16:101–17. 10.1214/ss/1009213286

[30] Fasiolo M Wood SN Zaffran M Nedellec R Goude Y. Fast calibrated additive quantile regression. J Am Stat Assoc. (2021) 116:1402–12. 10.1080/01621459.2020.1725521

[31] Merle R Busse M Rechter G Meer U. Regionalisation of Germany by data of agricultural structures. Berl Munch Tierarztl Wochenschr. (2012) 125:52–9. 10.2376/0005-9366-125-52 22372325

[32] Medrano-Galarza C LeBlanc SJ Jones-Bitton A DeVries TJ Rushen J Marie de Passillé A. Associations between management practices and within-pen prevalence of calf diarrhea and respiratory disease on dairy farms using automated milk feeders. J Dairy Sci. (2018) 101:2293–308. 10.3168/jds.2017-13733 29290433

[33] Gulliksen SM Lie KI Østerås O. Calf health monitoring in Norwegian dairy herds. J Dairy Sci. (2009) 92:1660–9. 10.3168/jds.2008-1518 19307648

[34] USDA. Dairy 2014, “Health and Management Practices on U.S. Dairy Operations, 2014” USDA–APHIS–VS–CEAH–NAHMS. Fort Collins, CO:USDA (2018).

[35] McGuirk SM. Disease management of dairy calves and heifers. Vet Clin North Am Food Anim Pract. (2008) 24:139–53. 10.1016/j.cvfa.2007.10.003 18299036 PMC7135781

[36] Olson KM Cassell BG McAllister AJ Washburn SP. Dystocia, stillbirth, gestation length, and birth weight in Holstein, Jersey, and reciprocal crosses from a planned experiment. J Dairy Sci. (2009) 9212:6167–75. 10.3168/jds.2009-2260 19923620

[37] Svensson C Hultgren J Oltenacu PA. Morbidity in 3–7-month-old dairy calves in south-western Sweden, and risk factors for diarrhoea and respiratory disease. Prev Vet Med. (2006) 74:162–79. 10.1016/j.prevetmed.2005.11.008 16406117

[38] Reis A Lopes CTdA Cerqueira VD Oliveira CMC Duarte MD. Navel ill of dairy calves from northeastern Pará Ciên. Anim Bras. (2009) 10 (Suppl 1):29–34.

[39] Steerforth DD Van Winden S. Development of clinical sign-based scoring system for assessment of omphalitis in neonatal calves. Vet Rec. (2018) 182:549. 10.1136/vr.104213 29459488

[40] Miessa LC Silva AA Botteon RCCM Botteon PTL. Morbidity and mortality by umbilical cord inflammation in dairy calves. Morbidade e mortalidade de bezerros leiteiros devido a processos inflamatórios do cordão umbilical. Hora Vet. (2003) 23:16–8.

[41] Thomas GW Jordaan P. Pre-slaughter mortality and post-slaughter wastage in bobby veal calves at a slaughter premises in New Zealand. N Z Vet J. (2013) 61:127–32. 10.1080/00480169.2012.734374 23181407

[42] Gulliksen SM Jor E Lie KI Hamnes IS Løken T Åkerstedt J. Enteropathogens and risk factors for diarrhea in Norwegian dairy calves. J Dairy Sci. (2009) 92:5057–66. 10.3168/jds.2009-2080 19762824 PMC7094401

[43] Gulliksen SM Jor E Lie KI Løken T Åkerstedt J Østerås O. Respiratory infections in Norwegian dairy calves. J Dairy Sci. (2009) 92:5139–46. 10.3168/jds.2009-2224 19762832 PMC7126448

[44] Hamnes IS Gjerde B Robertson L. Prevalence of Giardia and Cryptosporidium in dairy calves in three areas of Norway. Vet Parasitol. (2006) 140:204–16. 10.1016/j.vetpar.2006.03.024 16647210

[45] Klein-Jöbstl D Iwersen M Drillich M. Farm characteristics and calf management practices on dairy farms with and without diarrhea: a case-control study to investigate risk factors for calf diarrhea. J Dairy Sci. (2014) 97:5110–9. 10.3168/jds.2013-7695 24881793 PMC7094405

[46] Kaske M Leister T Smolka K Andresen U Kunz HJ Kehler W. Die neonatale Diarrhoe des Kalbes - IV Mitteilung: Kälberdurchfall als Bestandsproblem: Die Bedeutung der Kolostrumversorgung. Prakt Tierarzt. (2009) 90:756–67.

[47] Kashyap GDJA War ZA Gupta D Sahu N Singh S Kamdi B. Bacteriological and pathological study of a case of navel ill and its complications. J Immunol Immunopathol. (2018) 20:52–5. 10.5958/0973-9149.2018.00008.4

[48] Naik GAK Kavitha Rani B Kotresh A. Navel ill in new born calves and its successful treatment. Vet World. (2011) 4:326–7. 10.5455/vetworld.4.326

[49] Johnson KF Chancellor N Wathes DC. A cohort study risk factor analysis for endemic disease in pre-weaned dairy heifer calves. Animals. (2021) 11:378. 10.3390/ani11020378 33540923 PMC7913234

[50] Lorenz I Mee J Earley B More S. Calf health from birth to weaning. I General aspects of disease prevention. Ir Vet J. (2011) 64:10. 10.1186/2046-0481-64-10 21923898 PMC3184620

[51] Mee JF. Managing the calf at calving time. Am Assoc Bovine Pract Proc Ann Conf . (2008) 46–53. 10.21423/aabppro20084365

[52] Grover WM Godden S. Efficacy of a new navel dip to prevent umbilical infection in dairy calves. Bovine Practioner. (2011) 45:70–7. 10.21423/bovine-vol45no1p70-77

[53] Losinger WC Heinrichs AJ. Management variables associated with high mortality rates attributable to respiratory tract problems in female calves prior to weaning. J Am Vet Med Assoc. (1996) 209:1756–9. 8921037

[54] Pempek JA Schuenemann GM Holder E Habing GG. Dairy calf management - a comparison of practices and producer attitudes among conventional and organic herds. J Dairy Sci. (2017) 100:8310–21. 10.3168/jds.2017-12565 28822543

[55] Svensson C Liberg P. The effect of group size on health and growth rate of Swedish dairy calves housed in pens with automatic milk-feeders. Prev Vet Med. (2006) 73:43–53. 10.1016/j.prevetmed.2005.08.021 16191449

[56] Bartels CJM Holzhauer M Jorritsma R Swart WAJM Lam TJGM. Prevalence, prediction and risk factors of enteropathogens in normal and non-normal faeces of young Dutch dairy calves. Prev Vet Med. (2010) 93:162–9. 10.1016/j.prevetmed.2009.09.020 19819574 PMC7125667

[57] Izzo M Kirkland P Mohler V Perkins N Gunn A House J. Prevalence of major enteric pathogens in Australian dairy calves with diarrhoea. Aust Vet J. (2011) 89:167–73. 10.1111/j.1751-0813.2011.00692.x 21495987 PMC7159393

[58] Uhde FL. Prevalence of four enteropathogens in the faeces of young diarrhoeic dairy calves in Switzerland. Vet Record. (2008) 163:362–6. 10.1136/vr.163.12.362 18806281

[59] Pardon B Hostens M Duchateau L Dewulf J De Bleecker K Deprez P. Impact of respiratory disease, diarrhea, otitis and arthritis on mortality and carcass traits in white veal calves. BMC Vet Res. (2013) 9:79. 10.1186/1746-6148-9-79 23587206 PMC3639957

[60] Lundborg GK Oltenacu PA Maizon DO Svensson EC Liberg PGA. Dam-related effects on heart girth at birth, morbidity and growth rate from birth to 90 days of age in Swedish dairy calves. Prev Vet Med. (2003) 60:175–90. 10.1016/S0167-5877(03)00106-5 12900157

[61] Gulliksen SM Lie KI Sølverød L Østerås O. Risk factors associated with colostrum quality in Norwegian dairy cows. J Dairy Sci. (2008) 91:704–12. 10.3168/jds.2007-0450 18218758

[62] Johnsen JF Viljugrein H Bøe KE Gulliksen SM Beaver A Grøndahl AM. A cross-sectional study of suckling calves' passive immunity and associations with management routines to ensure colostrum intake on organic dairy farms. Acta Vet Scand. (2019) 61:7. 10.1186/s13028-019-0442-8 30700306 PMC6354394

[63] Maddox-Hyttel C Langkjær RB Enemark HL Vigre H. Cryptosporidium and Giardia in different age groups of Danish cattle and pigs—occurrence and management associated risk factors. Vet Parasitol. (2006) 141:48–59. 10.1016/j.vetpar.2006.04.032 16797848

[64] Rassel M Mishra P Rahman M Alam M. Exploring bacterial pathogens and risk factors associated with the occurrence of navel ill in calves. J Istanbul Vet Sci. (2020) 4:37–42. 10.30704/http-www-jivs-net.722788

[65] Renaud DL Kelton DF LeBlanc SJ Haley DB Duffield TF. Calf management risk factors on dairy farms associated with male calf mortality on veal farms. J Dairy Sci. (2018) 101:1785–94. 10.3168/jds.2017-13578 29248230

[66] Jansen J Lam TJGM. The role of communication in improving udder health. Vet Clin Food Anim Pract. (2012) 28:363–79. 10.1016/j.cvfa.2012.03.003 22664213

[67] Winder CB Bauman CA Duffield TF Barkema HW Keefe GP Dubuc J. Canadian national dairy study: heifer calf management. J Dairy Sci. (2018) 101:10565–79. 10.3168/jds.2018-14680 30172400

[68] Ellis-Iversen J Cook AJC Watson E Nielen M Larkin L Wooldridge M. Perceptions, circumstances and motivators that influence implementation of zoonotic control programs on cattle farms. Prev Vet Med. (2010) 93:276–85. 10.1016/j.prevetmed.2009.11.005 19963291

