# Supplementary material for: Benchmarking calf health: Assessment tools for dairy herd health consultancy based on reference values from 730 German dairies with respect to seasonal, farm type, and herd size effects
Source: Front Vet Sci. 2022 Sep 23;9:990798. doi: 10.3389/fvets.2022.990798 (PMC9539667; doi:10.3389/fvets.2022.990798)
Supplement: Supplementary file 1 [file Table_1.pdf]

Supplement

Benchmarking Calf Health: Assessment Tools for Dairy Herd Health Consultancy based on Reference Values from 730 German Dairies with Respect to Seasonal, Farm Type and Herd Size Effects.

DOI: 10.3389/fvets.2022.990798

Characterization of the three Regions (North, East, South).

The three selected regions were different in herd size, structure, and management. In the north, the farms had a median of 90 dairy cows (Interquartile Range, IQR: 59.0 - 129.0) and were often family businesses partly with external workers. Almost all farms were run as full-time businesses (99.2%). A farm in the northern region cultivated in the median an area of 100.0 hectares, thereof one half as grassland (pasture, roughage) and one half as arable land. Farms in the eastern region were large-scale agricultural enterprises, with a median of 251 dairy cows (IQR: 137.0 - 445.0) and external workers. These farms have historically grown from the former agriculture production cooperatives of the German Democratic Republic. A farm in the eastern region had a median area of 760.0 hectares in cultivation, thereof the largest part used as arable land (median of 536.5 hectares). In the eastern region, the farms often used veterinary services for health monitoring of the dairy herd (61.8%) and especially of youngstock (44.1 %). The southern region was characterized by small (median: 39.0 dairy cows), traditionally managed family businesses, some run as part-time businesses (19.5%). A farm in the southern region cultivated a median of 43.0 (IQR: 30.0 - 60.0) hectares, predominantly as grassland (22.0 hectares). The largest proportion of organic farms were found in the southern region (13.0%, details shown in Table 1).

Table S1: Study population and farm data for 730 farms in three regions (North, East, South) of Germany.

	Median (IQR)*			
	Overall	North	East	South
**Study population**				
dairy cows	84.0 (44.0, 188.0)	90.0 (59.0, 129.0)	251.0 (137.0, 445.0)	39.0 (26.0, 59.0)
preweaned calves on farm	13.0 (7.0, 27.0)	13.0 (9.0, 21.0)	37.0 (19.5, 70.5)	7.0 (4.0, 12.0)
preweaned calves examined	12.0 (6.0, 25.0)	12.0 (7.0, 19.0)	33.0 (17.0, 42.0)	7.0 (3.0, 11.0)
age at weaning (wk)	11.0 (10.0, 12.0)	10.0 (9.0, 12.0)	11.0 (10.0, 12.0)	11.0 (9.0, 12.0)
**Area under cultivation**				
total area (ha)	100.0 (52.0, 327.5)	100.0 (71.0, 129.0)	760.0 (320.0; 1,455.0)	43.0 (30.0, 60.0)
thereof grassland (ha)	44.0 (24.0, 100.0)	46.0 (30.0, 65.0)	162.5 (75.8, 300.0)	22.0 (15.0, 33.8)
thereof arable (ha)	50.0 (18.9, 200.0)	45.0 (25.0, 75.0)	536.5 (182.5, 1,150.0)	15.0 (3.0, 36.5)

*Interquartile Range.

Table S2. Farm organization and veterinary herd health management (VHHM) on 730 dairy farms in Germany in three regions (North, East, South).

	n (%)			
**Farm organization**	Overall	North	East	South
full-time business	676.0 (92.6)	235.0 (99.2)	243.0 (98.4)	198.0 (80.5)
part-time business	54.0 (7.4)	2.0 (0.8)	4.0 (1.6)	48.0 (19.5)
conventional	661.0 (90.4)	226.0 (95.4)	226.0 (91.5)	209.0 (84.6)
organic	64.0 (8.8)	11.0 (4.6)	21.0 (8.5)	32.0 (13.0)
transition[#]	6.0 (0.8)	0.0 (0.0)	0.0 (0.0)	6.0 (2.4)
**VHHM**				
for dairy cows	330.0 (45.3)	132.0 (55.7)	152.0 (61.8)	46.0 (18.7)
for youngstock	159.0 (21.8)	39.0 (16.5)	109.0 (44.1)	11.0 (4.5)

[#]Farms in process of transition from conventional to organic farming (in the analysis they are evaluated as conventional farms).

Table S3: Study population and farm data for 730 dairy farms in Germany stratified by herd size and farm type.

		Median (Min; Max)#						
	Total	Conventional						Organic
		Overall	1 - 40*	41 - 60*	61 - 120*	121 - 240*	> 241*	
**No. farms (n)**	**(n = 730)**	**(n = 666)**	**(n = 130)**	**(n= 99)**	**(n = 180)**	**(n = 119)**	**(n = 138)**	**(n = 64)**
**Study population**								
Dairy cows (n)	84 (1; 2,821)	90 (1; 2,821)	27 (1; 40)	51 (41; 60)	85 (61; 120)	162 (121; 239)	426 (241; 2,821)	42 (1; 297)
Preweaned calves (n)	14 (1; 350)	15 (1; 350)	5 (1; 19)	10 (1; 38)	13 (2, 47)	24 (3; 64)	66 (6; 350)	7 (1; 33)
Examined calves (n)	12 (1; 75)	13 (1; 75)	5 (1; 19)	9 (1; 28)	12 (2; 46)	21 (3; 63)	41 (5; 75)	6 (1; 33)
Examined calves (%)	96 (18; 100)	95 (82, 100)	100 (46; 100)	100 (42; 100)	100 (36; 100)	94 (43; 100)	79 (18; 100)	100 (42; 100)
**Area under Cultivation**								
Total area (ha)	100 (0; 7,000)	104 (0; 700)	32 (1; 114)	56 (0; 570)	92 (33; 1,600)	190 (65; 5,000)	1,300 (140; 7,000)	63 (12; 1,800)
Thereof grassland (ha)	45 (0; 2,900)	45 (0; 2,900)	18 (1; 62)	25 (0; 180)	41(0; 400)	78 (6; 600)	280 (5; 2,900)	40 (3; 650)
Thereof arable (ha)	50 (0; 6,600)	55 (0; 6,600)	10 (0; 84)	30 (0; 400)	45 (0; 1,200)	140 (0; 4,400)	960 (0; 6,600)	24 (0; 1,350
